# The Impact of the COVID-19 Pandemic on Tuberculosis Case Notification and Treatment Outcomes in Eswatini

**DOI:** 10.3389/ijph.2022.1605225

**Published:** 2022-10-26

**Authors:** Hloniphile Victory Masina, I-Feng Lin, Li-Yin Chien

**Affiliations:** ^1^ International Health Program, National Yang Ming Chiao Tung University, Taipei, Taiwan; ^2^ Institute of Public Health, National Yang Ming Chiao Tung University, Taipei, Taiwan; ^3^ Institute of Community Health Care, College of Nursing, National Yang Ming Chiao Tung University, Taipei, Taiwan

**Keywords:** COVID-19, tuberculosis, interrupted time series, retrospective cohort study, Eswatini

## Abstract

**Objectives:** We investigated the impact of COVID-19 on tuberculosis (TB) case notification and treatment outcomes in Eswatini.

**Methods:** A comparative retrospective cohort study was conducted using TB data from eight facilities. An interrupted time series analysis, using segmented Poisson regression was done to assess the impact of COVID-19 on TB case notification comparing period before (December 2018-February 2020, *n* = 1,560) and during the pandemic (March 2020–May 2021, *n* = 840). Case notification was defined as number of TB cases registered in the TB treatment register. Treatment outcomes was result assigned to patients at the end of treatment according to WHO rules.

**Results:** There was a significant decrease in TB case notification (IRR 0.71, 95% CI: 0.60–0.83) and a significant increase in death rate among registrants during the pandemic (21.3%) compared to pre-pandemic (10.8%, *p* < 0.01). Logistic regression indicated higher odds of unfavorable outcomes (death, lost-to-follow-up, and not evaluated) during the pandemic than pre-pandemic (aOR 2.91, 95% CI: 2.17–3.89).

**Conclusion:** COVID-19 negatively impacted TB services in Eswatini. Eswatini should invest in strategies to safe-guard the health system against similar pandemics.

## Introduction

Tuberculosis (TB) remains one of the leading cause of death globally. In 2018, 10 million TB cases and more than 1.5 million deaths were reported. Majority of the reported cases are from sub-Saharan Africa, a region that is also engulfed by HIV [1]. Great strides have been made in the fight against TB in the past decade. However, the emergence of COVID-19 on the already overwhelmed health care system has affected TB case notification and treatment outcomes, setting back the achievements made in the fight against TB [2]. COVID-19 has become a major health calamity, and a year after its emergence, it had already surpassed TB as the leading infectious disease [2]. The effects of COVID-19 have been experienced even in countries with state-of-the-art technologies and reasonable amount of human resource [3].

Eswatini have a high annual TB incidence at 309/100,000 and the highest HIV prevalence in the world at 27% among 15–49 years old [4–6]. Active case finders (ACFs) were introduced in 2016 and their main role was to find and diagnose TB cases in the community through visiting contacts of those already diagnosed, an exercise that helped increase the proportion of TB cases diagnosed [7]. The TB treatment success rate in Eswatini was reported to be at 86% in the year 2019, 4% less than the global target of 90% which the country is working towards achieving [8]. Through the introduction of advanced diagnostic techniques, like the GeneXpert, confirmatory diagnosis greatly improved. The number of cases notified and enrolled on TB treatment have been steadily declining at an average of 18%–15% per annum in Eswatini since 2010 [9, 10]. In 2018, a total of 3151 TB cases were notified in the country and 1900 cases were reported by the end of 2021 [9]. Studies have predicted a worrying possibility of TB case notifications declining in an alarming rate due to COVID-19 restrictions [11]. Other studies have predicted that, where COVID-19 hits areas with a high TB burden, TB associated deaths are anticipated to rise by at least 20% [12].

The impact of COVID-19 on TB has not been fully reported in sub-Saharan African countries [13]. Where there are studies done, the observation period was either short or the sample size too small [14]. It is in that regard that we intend to evaluate the impact of the COVID-19 pandemic on TB services by mainly focusing on the TB case notification and treatment outcomes among TB patients in Eswatini, a comparitive analysis for the years 2018–2019 (before COVID-19) and the years 2020–2021 (during COVID-19).

## Methods

### Country Background

Eswatini is a lower-middle-income country with a population of 1.2 million. Poverty levels are high at 63%, and 77% of the country’s population resides in rural areas [15]. The country is divided into four geographic regions namely Hhohho, Manzini, Shiselweni and Lubombo. TB care is offered in three levels: primary, secondary and tertiary by primary health care facilities (referred to as small or baby clinics), health centers, and referrals (hospitals), respectively. Health centers offers both primary and secondary TB care while the referrals offer tertiary care. Most primary health care facilities offer TB screening and then refer to health centers or referrals for further diagnosis and treatment. A total of 157 health facilities provide TB treatment in Eswatini, 4 of which are referrals (one in each region).

### Design and Participants

This was a retrospective cohort study that used an interrupted time series design to compare outcomes before and during the pandemic. The study collected paper-based data from TB registers and directly observed cards from December 2018 to May 2021. Ethical approval was granted by the Eswatini Health and Human Research Review Board (EHHRRB047/2021). Data was collected from 8 purposively selected health care facilities, the referral and the health center with the highest number of TB cases in the region, two from each of the four regions of Eswatini. We focused on health centers and referrals because they have the greatest number of TB patients in the country and they offer comprehensive TB care and management.

A total of 2,400 registrants’ records were extracted and analysed for the TB case notification. A total of 2,294 registrants’ records were analysed for treatment outcomes, excluding those with missing information on study variables (*n* = 106 or 4%).

### Measurements

This study had two outcomes. The first outcome was TB case notification, defined as the number of TB cases registered in the facility TB treatment registers. The second outcome was TB treatment outcomes, a binary variable; unfavorable (death, lost-to-follow-up, not-evaluated) and favorable treatment outcomes (cured and treatment completed) [16, 17]. Cured was defined as a confirmed TB patient becoming smear or culture negative in the last month of treatment, treatment completed being a patient who successfully completes treatment with no record of a negative smear, died defined as a TB patient who dies for any reason during the course of treatment, lost to follow up being a patient whose treatment was interrupted for two consecutive months or more, and not evaluated was defined as a patient with no assigned treatment outcome. None of the participants were documented as having treatment failure. This is so because of the improved accessibility to drug susceptibility tests which ensures that TB patients are initially put on the right treatment. The facility that initiates the treatment is responsible for tracking and updating the registrants’ treatment outcome even if the patients were transferred out of the facility during treatment.

Other variables included patient’s demographic data; age was categorised into ≤14, 15–24, 25–44, 45–64, and ≥65, sex was categorised as male and female; residence as rural and urban. Other covariates included HIV status categorised as negative or positive. For anti-retroviral therapy (ART) status, participants were either on ART or not. For the TB diagnosis status, it was either “bacteriologically confirmed or not (clinically diagnosed)”, TB history was either “new or previously treated”, case finding site was either “health facility or community.” Patient’s data on comorbidities (diabetes or hypertension) was also collected. Lastly, the “type of TB” was categorised into drug susceptible TB and multi-drug resistant TB.

### Data Analysis

Data was analysed using Stata 15. Data distribution were explored using frequencies and proportions. Chi squared test was used to explore categorical variables. To answer the objective on TB case notification, we undertook an interrupted time series analysis using segmented Poisson regression model with Newey–West standard errors which help control for autocorrelation and heteroscedasticity [18]. For comparison purposes, we defined the period December 2018-February 2020 as the “pre-COVID-19” era and March 2020-May 2021 as “during COVID-19,” putting the “interruption” at the beginning of 2020 (March 2020), which is the time when COVID-19 was introduced into the population of Eswatini. We reported incidence rate ratios (IRR), which we obtained by exponentiating the Poisson regression coefficients and are interpreted as the ratio of slopes in the two time periods.

Binary logistic regression model was used to examine the differences in treatment outcomes in the two observational periods. For comparison purposes, treatment outcomes were compared pre (Dec 2018-Feb 2020) and during COVID-19 (March 2020-May 2021) and adjusted odds ratios (aOR), 95% confident intervals (95% CI) and *p*-values were reported.

## Results

Table 1 shows descriptive statistics stratified by the time period (pre and during COVID-19). A total of 2400 TB participants were notified during the entire study period where 1,560 were notified prior to COVID-19 and 840 were notified during the COVID-19.3.5% (*n* = 84) of the total participants were children <14 years of age. There were no significant differences in the age groups distribution before or during the pandemic. Over half of the participants were from Hhohho region both pre (52.1%) and during COVID-19 (61.3%). There was a decrease in proportions of patients from Manzini region from pre (21.8%) to during COVID-19 (14.0%; *p* < 0.01).

**TABLE 1:** Descriptive statistics among tuberculosis patients in Eswatini having stratified by the time period (before pandemic 2018–2019 vs. during pandemic 2020–2021) (*N* = 2,400) (TB Registration Study, Eswatini, 2021–2022).

Variables	Total n (%)	Time period		
		Pre-COVID (December 2018–February 2020) *n* = 1,560	Intra-COVID (March 2020–May 2021) *n* = 840	*p*-value
Treatment outcomes				*<0.01
Treatment Completed	813 (34.1)	540 (34.3)	273 (33.5)	
Cured	1,200 (50.3)	847 (53.8)	353 (43.4)	
Deaths	343 (14.4)	170 (10.8)	173 (21.3)	
Lost to follow up	23 (1.0)	11 (0.7)	12 (1.5)	
Not evaluated	9 (0.4)	6 (0.4)	3 (0.4)	
Age group				0.09
≤14	84 (3.5)	59 (3.7)	25 (3.1)	
15–24	301 (12.6)	216 (13.7)	85 (10.5)	
25–44	1,365 (57.1)	900 (57.0)	465 (57.3)	
45–64	511 (21.4)	324 (20.5)	187 (23.0)	
≥65	129 (5.4)	79 (5.0)	50 (6.2)	
Gender				0.64
Male	941 (39.3)	628 (39.6)	313 (38.6)	
Female	1,456 (60.7)	958 (60.4)	498 (61.4)	
Residential Location				0.06
Rural	1,604 (67.3)	1,077 (68.6)	527 (64.7)	
Urban	781 (32.8)	494 (31.4)	287 (35.3)	
Region				*<0.01
Hhohho	1,325 (55.2)	826 (52.1)	499 (61.3)	
Lubombo	284 (11.8)	195 (12.3)	89 (10.9)	
Manzini	459 (19.1)	345 (21.8)	114 (14.0)	
Shiselweni	332 (13.8)	220 (13.9)	112 (13.8)	
HIV status				0.84
Positive	1,584 (66.0)	1,049 (66.1)	535 (65.7)	
Negative	816 (34.0)	537 (33.9)	279 (34.3)	
ARV status among HIV positive individuals (*n* = 1,584)				*<0.01
Not on ART	25 (1.6)	9 (0.9)	16 (3.0)	
On ART	1,559 (98.4)	1,040 (99.1)	519 (97.0)	
History of TB				0.28
New	2,107 (87.9)	1,383 (87.4)	724 (88.9)	
Previously treated	289 (12.1)	199 (12.6)	90 (11.1)	
Type of TB				*<0.01
DRTB	182 (7.6)	140 (8.9)	42 (5.2)	
DSTB	2,213 (92.4)	1,442 (91.2)	771 (94.8)	
Case finding site				*<0.01
Community	151 (6.3)	151 (9.5)	0 (0.0)	
Facility	2,249 (93.7)	1,435 (90.5)	814 (100.0)	
Comorbidities (diabetes or hypertension)				0.29
Present	159 (6.6)	99 (6.2)	60 (7.4)	
Not present	2,241 (93.4)	1,487 (93.8)	754 (92.6)	
Bacteriologically confirmed TB				0.16
Yes	1,318 (55.5)	882 (56.6)	436 (53.6)	
No	1,055 (44.5)	677 (43.4)	378 (46.4)	

*p-value <0.05; ARV-anti retroviral; ART-anti retroviral therapy; DRTB- drug resistant TB; DSTB-drug susceptible TB. Number of participants with Missing: Age 10, gender 3, ARV status 30, residential location 15, bacteriologically confirmed 27, treatment outcome 12, type of TB 5, history of TB 4.

The proportion of “cured” registrants were higher (53.8%) before the pandemic compared to during the pandemic (43.4%). Death rate was significantly higher (*p* < 0.01) in the pandemic (21.3%) than prior (10.8%) and so was the lost to follow up rate (1.5%, 0.7%). While 9.5% of the registrants were found through community case finding before the pandemic, none were found through community during the pandemic.

**FIGURE 1:**
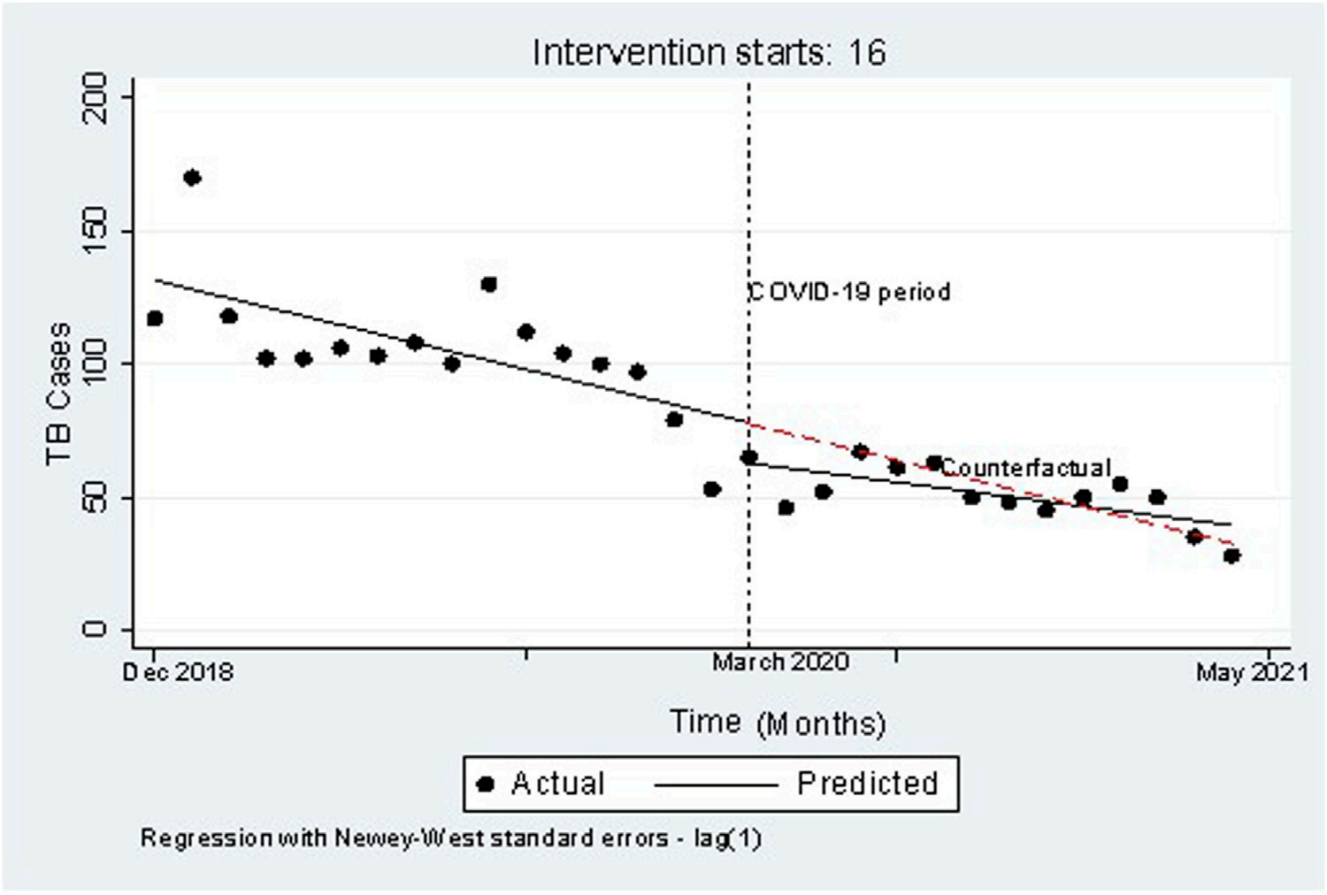
Twoway graph of the number of tuberculosis cases notified before (2018–2019) and during (2020–2021) COVID-19 among tuberculosis patients in Eswatini against months (*n* = 2,400) (TB Registration Study, Eswatini, 2021–2022).

Figure 1 is a graphic description of the number of TB cases reported before and during the pandemic (*N* = 2,400) and the pandemic having been introduced in Eswatini in March of 2020. The two-way graph shows a sudden drop in the number of TB cases simultaneous with the introduction of the pandemic into the population. However, the observed slope is not as steep as the predicted slope following the sudden drop, suggesting a gradual increase in observed number of cases in comparison with predicted numbers, and finally showed the crossing of the predicted and observed lines.

Table 2 is the segmented Poisson regression of notified TB cases before and during the pandemic. Model 1 shows the effect of the pandemic on TB treatment outcomes comparing to pre-COVID, the estimated IRR of reporting TB cases decreased by 0.47 (95% CI: 0.39–0.55) during the pandemic. After controlling for seasonality (Model 2) the estimated IRR of reporting TB cases remained being 0.47 (95% CI: 0.40–0.55) in the time of the pandemic relative to the period prior to the pandemic. In model 3, after having controlled for declining time trend of TB case notification additionally, the IRR decreased by a factor of 0.71 (95% CI: 0.60–0.83).

**TABLE 2:** Segmented Poisson regression of notified tuberculosis cases before (2018–2019) and during (2020–2021) COVID-19 in Eswatini (*N* = 2,400) (TB Registration Study, Eswatini, 2021–2022).

Variables	Model 1		Model 2		Model 3	
	IRR (95%CI)	*p*-value	IRR (95%CI)	*p*-value	IRR (95%CI)	*p*-value
COVID-19 time period (ref pre-COVID-19)		1				
Intra-COVID	0.47 (0.39–0.55)	*<0.01	0.47 (0.40–0.55)	*<0.01	0.71 (0.60–0.83)	*<0.01
Seasonality (ref Summer)						
Winter			0.94 (0.82–1.07)	0.36	0.90 (0.83–0.98)	*0.01
Time trend					0.97 (0.96–0.98)	*<0.01

*p-value <0.05.

Logistic regression analysis (Table 3) shows the results of the impact of the COVID-19 pandemic on TB treatment outcomes. Patients who were treated during the pandemic had higher odds of experiencing unfavorable TB treatment outcomes compared to those treated before the pandemic even after controlling for confounders (aOR 2.91, 95% CI: 2.17–3.89). Age ≥45 years (aOR 2.75, 95% CI: 1.14–6.23 for 45–64 years of age; aOR 4.57, 95% CI: 1.79–11.65 for ≥65 years), having HIV (aOR 1.50, 95% CI: 1.02–2.20), comorbid with diabetes or hypertension (aOR 2.92, 95% CI:1.84–4.62), case finding site being a facility (aOR 1.97,95% CI: 1.06–3.64), and being clinically diagnosed without a bacteriologically confirmed test result (aOR 1.79, 95% CI: 1.33–2.42) were factors that were found to be associated with poor treatment outcomes.

**TABLE 3:** Logistic regression of the association between the time period and unfavorable treatment outcomes among people on TB treatment in Eswatini (*n* = 2,294§) (TB Registration Study, Eswatini, 2021–2022).

Variables	Model 1		Model 2	
	OR (CI)	*p*-value	OR (CI)	*p*-value
Time period (ref pre-COVID-19)	2.81 (2.22–3.57)	*<0.01	2.91 (2.17–3.89)	*<0.01
Age group (ref ≤ 14)				
15–24			1.58 (0.63–3.95	0.33
25–44			1.52 (0.64–3.61)	0.34
45–64			2.75 (1.14–6.23)	0.02
≥65			4.57 (1.79–11.65)	*<0.01
Gender (ref Male)				
Female			1.00 (0.78–1.29)	1.00
HIV status (ref Negative)				
Positive			1.50 (1.02–2.20)	*0.04
ART status (ref on ART)				
Not on ART			1.00 (0.68–1.47)	0.99
Location (ref Rural)				
Urban			0.96 (0.74–1.26)	0.79
History of TB (ref New)				
Previously treated			0.82 (0.56–1.20)	0.30
Type of TB (ref DRTB)				
DSTB			0.68 (0.43–1.07)	0.10
Case Finding site (ref Community)				
Health facility			1.97 (1.06–3.64)	*<0.01
Comorbidities (diabetes or hypertension; ref None)				
Present			2.92 (1.84–4.62)	*<0.01
Bacteriologically confirmed TB (ref Yes)				
No			1.79 (1.33–2.42)	*<0.01

*p-value <0.05; ART-anti retroviral therapy; DRTB- drug resistant TB; DSTB-drug susceptible TB; §, Complete case analysis.

## Discussion

In this study we have found a significant drop (29%) in the TB case notification number in the course of the COVID-19 pandemic after having accounted for time varying and other factors (autocorrelation, seasonality, heteroscedasticity, and time trend). The major plunge in TB case notification for the time of the COVID-19 pandemic might be due to two possible explanations. One possibility is that fewer TB cases were identified due to reduced case finding and public anxiety about seeking healthcare. Alternatively, there may have been an actual reduction in TB infections due to public health restrictions. The reduced number of TB notifications could reflect decreased transmission associated with physical distancing and the increased use of face masks as recent analysis proposed that physical distancing decreases transmission of TB by 10% in high TB burden countries [19, 20]. Our findings, however showed a 29% decrease, a magnitude far greater than the 10% and therefore we believe part of it could be from fewer infection, but more is from the malfunction of the service and decreased accessibility to health services in the pandemic. In addition, from the graphical results (Figure 1), we feel that the first explanation is more likely since the decrease in case notification was more evident in the early pandemic period, and the number of case notification overlapped with the pre-COVID time trend in the late period.

TB cases that were identified and screened in the community were most likely missed during the COVID-19 due to the suspension of ACFs, as evidenced by Table 1 results which shows that zero cases were found through community active case finding during the pandemic. Studies have shown that ACFs lead to an increase in the overall TB case notification [21], and to suspend ACF will definitely negatively affect TB case notification. Besides reduced case finding, as COVID-19 spreads, prevalent fear of the coronavirus prevented people from pursuing medical care. Due to fear of being infected with COVID-19, fewer people would attend health facilities than usual [22–24]. Additionally, lockdown measures which were put in place restricting movement. In Kenya, 50% of TB diagnosed patients had difficulties accessing public transport to health facilities during the pandemic [25]. All those may have contributed to the decrease in TB case notification during the COVID-19 period.

In the living with COVID-19 era, we suggest that rather than suspending active case finding, in the future, it should be adapted to the COVID-19 situation while considering infection control measures [26]. In high TB prevalence settings, ACF should be provided to close contacts of confirmed TB cases by health workers using appropriate personal protective equipment and algorithms that enable physical distancing and reduced patient contact with health services [26, 27]. Besides, movement restrictions made it more difficult for people to physically access health services [25]. Government should take this into consideration when implement lockdown policies.

Further, our findings confirmed that patients had higher odds of experiencing unfavorable TB treatment outcomes during the pandemic. Similar findings were reported where it was found that COVID-19 has brought disruptions in the care and management of TB such that it led to an increase in unfavorable treatment outcomes [28, 29]. Probable explanations for the increase in unfavorable TB treatment outcomes includes lockdown rules and the fear of contracting COVID-19 in hospitals. In the era of COVID-19 routine health care services are no longer made priority and resources are diverted to COVID-19 activities [30]. To ensure continuity of TB services amid the COVID-19 threat, strategies like activating remote treatment support through the use of telemedicine, emphasizing community-based care through the use of TB survivors to provide care and support to those on treatment and through the sharing of innovative practices as studies on how to align TB services with the COVID-19 response are being done in several settings [31, 32]. The implementation of phone consultations can be one strategy to cushion TB services during emergency periods in Eswatini. Community based care, adapted to the COVID situation, can also be used to monitor adherence, ensure that patients with comorbidities are also closely monitored away from the health care system and prompt referral done in the case of emergencies [31].

Over the next few years, the country needs to create policies that promotes bi-directional screening, multi-pathogen tests and integration of the TB/COVID-19 surveillance to avoid diagnostic delays and improve treatment outcomes [33]. TB services should further be integrated into community ART services which have been well implemented in Eswatini before the pandemic although the extent in which the pandemic has affected community ART services has not been established.

Factors found to be associated with poor treatment outcomes during the pandemic were similar to what other studies have already described (age ≥45, having HIV, case finding site at a facility, having a clinical diagnosis without a bacteriologically confirmed result, and an existing comorbidity of diabetes or hypertension) [12, 28, 30]. Older age, being HIV positive and having an existing comorbidity were found to be factors mostly associated with death as an outcome among TB patients treated during the pandemic [17, 34]. Continuous efforts to prevent poor treatment outcomes among those high-risk individuals are needed.

Our study had some limitations. We used treatment register and therefore lost undiagnosed TB patients and patients diagnosed with TB prior to treatment initiation. Pre-treatment loss to follow-up may be common in Africa [35]. For treatment outcomes, 106 (4%) participants were excluded from the analysis due to missing values and this might have introduced bias. We combined death (*n* = 241), lost-to-follow-up (*n* = 15), and not-evaluated (*n* = 9) into unfavorable treatment outcomes to avoid small cell size in the analysis. Those with unfavorable treatment outcomes were mostly deaths. Therefore, factors associated with unfavorable treatment outcomes in the study were mainly due to their association with death. We also lacked information on other factors varying over time during the study period, e.g., GeneXpert stock outs periods and therefore we could not control for it. The purposive selection of study sites might have introduced bias. Data on treatment outcomes was collected during the two major COVID-19 waves in Eswatini, we are lacking information on the impact of the pandemic on the third and fourth waves. We cannot determine whether TB treatment outcomes died and lost to follow-up were directly related to TB or not due to a lack of information.

### Conclusion

COVID-19 had negatively impacted on TB case notification and treatment outcomes in Eswatini. Developing and strengthening strategies and policies to rapidly adapt TB control measures to the new challenge should be made priority so as to meet the End-TB strategy goal of 2035.

## References

[1] Mchunu G Van Griensven J Hinderaker S Kizito W Sikhondze W Manzi M High Mortality in Tuberculosis Patients Despite HIV Interventions in Swaziland. Public Health Action (2016) 6:105–10. 10.5588/pha.15.0081 27358803 PMC4913672

[2] Togun T Kampmann B Stoker NG Lipman M. Anticipating the Impact of the COVID-19 Pandemic on TB Patients and TB Control Programmes. Ann Clin Microbiol Antimicrob (2020) 19(1):21–6. 10.1186/s12941-020-00363-1 32446305 PMC7245173

[3] Núñez A Sreeganga S Ramaprasad A. Access to Healthcare during COVID-19. Int J Environ Res Public Health (2021) 18:2980. 10.3390/ijerph18062980 33799417 PMC7999346

[4] World Bank. Incidence of Tuberculosis (Per 100 000 People)-Eswatini (2022). Available from: https://data.worldbank.org/indicator/SH.TBS.INCD (Accessed April 4, 2022).

[5] United Nations Programme on HIV/AIDS. Swaziland Global AIDS Response Progress Reporting (2014). Available from: https://www.unaids.org/sites/default/files/media_asset/GARPR_2014_guidelines_en_0.pdf (Accessed March 31, 2022).

[6] Nkambule R Philip NM Reid G Mnisi Z Nuwagaba-Biribonwoha H Ao TT HIV Incidence, Viremia, and the National Response in Eswatini: Two Sequential Population-Based Surveys. PLoS One (2021) 16:e0260892. 10.1371/journal.pone.0260892 34855890 PMC8639055

[7] Kay AW Sandoval M Mtetwa G Mkhabela M Ndlovu B Devezin T Vikela Ekhaya: A Novel, Community-Based, Tuberculosis Contact Management Program in a High Burden Setting. Clin Infect Dis (2021) 74:1631. 10.1093/cid/ciab652 PMC9070808 34302733

[8] Centers for Disease and Control and Prevention (Cdc). Global HIV and TB: Eswatini Country Profile (2021). Available from: https://www.cdc.gov/globalhivtb/where-we-work/eswatini/eswatini.html (Accessed March 31, 2022).

[9] Tuberculosis in Eswatini. Tuberculosis in Eswatini (2020). Available from: https://www.stoptb.org/static_pages/SWZ_Dashboard.html (Accessed April 26, 2021).

[10] Kerschberger B Schomaker M Telnov A Vambe D Kisyeri N Sikhondze W Decreased Risk of HIV-Associated TB during Antiretroviral Therapy Expansion in Rural Eswatini from 2009 to 2016: a Cohort and Population-Based Analysis. Trop Med Int Health (2019) 24(9):1114–27. 10.1111/tmi.13290 31310029 PMC6852273

[11] Arega B Negesso A Taye B Weldeyohhans G Bewket B Negussie T Impact of COVID-19 Pandemic on TB Prevention and Care in Addis Ababa, Ethiopia: a Retrospective Database Study. BMJ Open (2022) 12:e053290. 10.1136/bmjopen-2021-053290 PMC8829833 35135769

[12] Manhiça I Augusto O Sherr K Cowan J Cuco RM Agostinho S COVID-19-related Healthcare Impacts: an Uncontrolled, Segmented Time-Series Analysis of Tuberculosis Diagnosis Services in Mozambique, 2017–2020. BMJ Glob Health (2022) 7:e007878. 10.1136/bmjgh-2021-007878 PMC9021460 35443938

[13] Fei H Yinyin X Hui C Ni W Xin D Wei C The Impact of the COVID-19 Epidemic on Tuberculosis Control in China. Lancet Reg Health West Pac (2020) 3:100032. 10.1016/j.lanwpc.2020.100032 34173601 PMC7511841

[14] Magro P Formenti B Marchese V Gulletta M Tomasoni LR Caligaris S Impact of the SARS-CoV-2 Epidemic on Tuberculosis Treatment Outcome in Northern Italy. Eur Respir J (2020) 56:2002665. 10.1183/13993003.02665-2020 32703780 PMC7377210

[15] Walker C Burtscher D Myeni J Kerschberger B Schausberger B Rusch B They Have Been Neglected for a Long Time”: a Qualitative Study on the Role and Recognition of Rural Health Motivators in the Shiselweni Region. Eswatini Hum Resour Health (2020) 18(1):1–9. 10.1186/s12960-020-00504-9 PMC7504860 32958066

[16] Chen H Zhang K. Insight into the Impact of the COVID-19 Epidemic on Tuberculosis burden in China. Eur Respir J (2020) 56:2002710. 10.1183/13993003.02710-2020 32703778 PMC7397949

[17] Nanzaluka FH Chibuye S Kasapo CC Langa N Nyimbili S Moonga G Factors Associated with Unfavourable Tuberculosis Treatment Outcomes in Lusaka, Zambia, 2015: a Secondary Analysis of Routine Surveillance Data. Pan Afr Med J (2019) 32:159. 10.11604/pamj.2019.32.159.18472 31308862 PMC6609856

[18] Bottomley C Scott JAG Isham V. Analysing Interrupted Time Series with a Control. Epidemiol Methods (2019) 8:1. 10.1515/em-2018-0010

[19] Quaife M Van Zandvoort K Gimma A Shah K McCreesh N Prem K The Impact of COVID-19 Control Measures on Social Contacts and Transmission in Kenyan Informal Settlements. BMC Med (2020) 18(1):316–1. 10.1186/s12916-020-01779-4 33012285 PMC7533154

[20] Kwak N Hwang S-S Yim J-J. Effect of COVID-19 on Tuberculosis Notification, South Korea. Emerg Infect Dis (2020) 26(10):2506–8. 10.3201/eid2610.202782 32672531 PMC7510739

[21] Beckert P Sanchez-Padilla E Merker M Dreyer V Kohl TA Utpatel C MDR *M. tuberculosis* Outbreak Clone in Eswatini Missed by Xpert Has Elevated Bedaquiline Resistance Dated to the Pre-treatment Era. Genome Med (2020) 12(1):104–11. 10.1186/s13073-020-00793-8 33239092 PMC7687760

[22] Talevi D Socci V Carai M Carnaghi G Faleri S Trebbi E Mental Health Outcomes of the CoViD-19 Pandemic. Riv Psichiatr (2020) 55(3):137–44. 10.1708/3382.33569 32489190

[23] Xiong J Lipsitz O Nasri F Lui LM Gill H Phan L Impact of COVID-19 Pandemic on Mental Health in the General Population: A Systematic Review. J Affect Disord (2020) 277:55–64. 10.1016/j.jad.2020.08.001 32799105 PMC7413844

[24] Yang J Kwon Y Kim J Jang Y Han J Kim D Delays in the Diagnosis and Treatment of Tuberculosis during the COVID-19 Outbreak in the Republic of Korea in 2020. Osong Public Health Res Perspect (2021) 12(5):293–303. 10.24171/j.phrp.2021.0063 34719221 PMC8561018

[25] Global Civil Society. The Impact of COVID-19 on the TB Epidemic: A Community Perspective. Geneva, Switzerland: Global Civil Society (2021).

[26] Chan G Triasih R Nababan B du Cros P Wilks N Main S Adapting Active Case-Finding for TB during the COVID-19 Pandemic in Yogyakarta, Indonesia. Public Health Action (2021) 11(2):41–9. 10.5588/pha.20.0071 34159059 PMC8202624

[27] World Health Organisation. Maintaining Essential Health Services: Operational Guidance for the COVID-19 Context: Interim Guidance (2020). Available from: https://apps.who.int/iris/handle/10665/332240 (Accessed April 20, 2022).

[28] Caren GJ Iskandar D Pitaloka DA Abdulah R Suwantika AA. COVID-19 Pandemic Disruption on the Management of Tuberculosis Treatment in Indonesia. J Multidiscip Healthc (2022) 15:175–83. 10.2147/JMDH.S341130 35115781 PMC8801372

[29] Chenciner L Annerstedt KS Pescarini JM Wingfield T. Social and Health Factors Associated with Unfavourable Treatment Outcome in Adolescents and Young Adults with Tuberculosis in Brazil: a National Retrospective Cohort Study. Lancet Glob Health (2021) 9(10):1380–90. 10.1016/s2214-109x(21)00300-4 34534486

[30] Loveday M Cox H Evans D Furin J Ndjeka N Osman M Opportunities from a New Disease for an Old Threat: Extending COVID-19 Efforts to Address Tuberculosis in South Africa. S Afr Med J (2020) 110(12):1160. 10.7196/SAMJ.2020.v110i12.15126 33403958

[31] World Health Organisation. COVID-19: Considerations for Tuberculosis (2021). Available from: https://apps.who.int/iris/handle/10665/341126?locale-attribute=es& (Accessed May 23, 2022).

[32] Shen X Sha W Yang C Pan Q Cohen T Cheng S Continuity of Services for Patients with Tuberculosis in China in the COVID-19 Era. medRxiv (2020).

[33] Bardhan M Hasan MM Ray I Sarkar A Chahal P Rackimuthu S Tuberculosis amidst COVID-19 Pandemic in India: Unspoken Challenges and the Way Forward. Trop Med Health (2021) 49(1):84–5. 10.1186/s41182-021-00377-1 34674772 PMC8528656

[34] Chung-Delgado K Guillen-Bravo S Revilla-Montag A Bernabe-Ortiz A. Mortality Among MDR-TB Cases: Comparison with Drug-Susceptible Tuberculosis and Associated Factors. PloS one (2015) 10(3):e0119332. 10.1371/journal.pone.0119332 25790076 PMC4366185

[35] MacPherson P Houben RM Glynn JR Corbett EL Kranzer K. Pre-treatment Loss to Follow-Up in Tuberculosis Patients in Low-And Lower-Middle-Income Countries and High-burden Countries: a Systematic Review and Meta-Analysis. Bull World Health Organ (2014) 92:126–38. 10.2471/BLT.13.124800 24623906 PMC3949536

